# Breakfast frequency and psychosomatic complaints among adolescents: a repeated cross-sectional analysis of the HBSC study

**DOI:** 10.1186/s13690-026-01914-2

**Published:** 2026-04-10

**Authors:** Yuzhong Duan, Jiao Yang, Dankang Li

**Affiliations:** 1 The Ninth Medical Center, Chinese PLA General Hospital, Beijing, 100101 People’s Republic of China; 2 Institute of Physical and Arts Education, China National Academy of Educational Sciences, Beijing, 100080 People’s Republic of China; 3 Department of Public Health and Preventive Medicine, School of Medicine, Jianghan University, Hubei 430056 Wuhan, People’s Republic of China; 4 Hubei Key Laboratory of Cognitive and Affective Disorders, Jianghan University, Hubei 430056 Wuhan, People’s Republic of China; 5 Hubei Provincial Demonstration Center for Experimental Medicine Education, School of Medicine, Jianghan University, Hubei 430056 Wuhan, People’s Republic of China

**Keywords:** Psychosomatic complaints, Breakfast consumption, Adolescent

## Abstract

**Background:**

To examine the non-linear association between breakfast consumption frequency and psychosomatic complaints among adolescents.

**Methods:**

Data were obtained from five waves (2002, 2006, 2010, 2014, and 2018) of the multi-country Health Behaviour in School-aged Children (HBSC) study. Psychosomatic complaints were assessed using eight items on psychosomatic complaints, combined into a composite score ranging from 0 to 32. Breakfast consumption frequency was measured by the number of days per week. Multilevel generalized additive models were applied to evaluate potential nonlinearity between breakfast frequency and psychosomatic complaints.

**Results:**

A total of 918,564 adolescents were included, with a mean (SD) age of 13.59 (1.64) years, of whom 473,633 (51.6%) were girls. Breakfast frequency showed a significant non-linear association to psychosomatic complaints (*P* for nonlinearity < 0.001). Compared with daily consumption (adjusted mean score: 7.41; 95% CI: 7.39–7.43), breakfast skipping was associated with a 2.84-point (β: 2.84, 95% CI: 2.75–2.93) higher symptom score (adjusted mean score: 10.17; 95% CI: 10.08–10.26). This difference corresponded to 8.9% of the scale range (POMP: 8.9%; 95% CI: 8.6–9.2) and a SMD of 0.49 (95% CI: 0.47–0.50). All association remained significant after False Discovery Rate correction (*P*-FDR < 0.001) and remained consistent across all survey years. Stratified analyses indicated that the association was stronger among females (β: 3.39, 95% CI: 3.26–3.52) than among males (β: 2.02, 95% CI: 1.90–2.15) and was more pronounced in higher grades (β: 2.07, 95% CI: 1.86–2.28 in grade 5; β: 2.88, 95% CI: 2.75–3.01 in grade 7; β: 2.98, 95% CI: 2.82–3.14 in grade 9).

**Conclusions:**

Breakfast frequency showed an inverse, non-linear association with psychosomatic complaints among adolescents, with consistent findings across survey waves. In addition, this association was more pronounced among females and adolescents in higher school grades.

**Supplementary Information:**

The online version contains supplementary material available at 10.1186/s13690-026-01914-2.

**Table:** 

Text box 1. Contributions to the literature
• Provides large-scale cross-national evidence on the nonlinear association between breakfast consumption frequency and adolescent psychosomatic Complaints.
• Identifies gender and school grade as key modifiers, with stronger adverse effects of skipping breakfast in girls and older adolescents.
• Highlights a strengthening association in more recent survey years, underscoring the growing public health importance of promoting regular breakfast consumption

## Background

Adolescence is considered a crucial phase for the development of mental health problems [1], and is also a period during which young people are most vulnerable to experiences of mental ill-health and the onset of mental disorders [2, 3]. Globally, approximately 14% of young people aged 10 to 19 experience mental health problems, which are among the leading causes of illness and disability in this group [4]. Identifying modifiable risks is therefore a public health priority.

Breakfast is one such behavior and has been linked to adolescent mental health [5, 6]. Studies from diverse settings report that skipping breakfast is associated with depression, anxiety, and psychological distress [7–9], while daily breakfast is associated with lower distress and better academic performance [10]. Yet policy-relevant evidence remains fragmented, as many studies are single-country or short-term, and definitions of breakfast [11–13] and mental health [14–16] vary across contexts, limiting cross-national comparison and obscuring how associations may change as food access and eating patterns evolve. Findings from 31 countries indicate that approximately one third of adolescents skip breakfast and that this behavior has become more common over time [17]. This trend underscores the need for harmonized, cross-country evidence over extended periods to clarify the association between breakfast consumption and psychosomatic complaints, identify priority groups, and inform education, outreach, and nutrition policy. Nevertheless, several key questions remain. (1) although a robust nonlinear dose-response relationship between BMI and psychosomatic complaints has been reported [18], whether breakfast frequency exhibits a similar non-linear association remains unknown. Identifying potential thresholds or points of diminishing returns may help inform population-level prevention strategies without implying causal effects. (2) as breakfast skipping has become more common, it is unclear whether its association with psychosomatic complaints differs across survey periods. (3) age and sex are well-established effect modifiers in the association between diet and mental health [19–23], whether the breakfast-psychosomatic complaints association varies by age and sex warrants further investigation.

To address these gaps, we used data from the Health Behaviour in School-aged Children (HBSC) study, a large repeated cross-national survey of adolescents from 45 countries and regions in Europe, Central Asia, and North America. HBSC uses harmonized measures of breakfast frequency and psychosomatic complaints across countries and survey years, enabling comparable estimates over time and place. Although these measures are necessarily coarse, their standardization facilitates cross-national and temporal comparisons. The primary objective of this study was to examine the non-linear association between breakfast frequency and psychosomatic complaints. We further assessed whether this association was stable across survey years and whether it was modified by gender and grade.

## Methods

### Data source and participants

This study is an observational repeated cross-sectional analysis based on the international Health Behaviour in School-aged Children (HBSC) study. The HBSC study is a World Health Organization Collaborative Cross-National Survey which has been conducted every 4 years since 1983 across Europe, North America, and the Middle East. HBSC collects data on the health and well-being, living environments, social relationships, and health behaviors of 11-, 13-, and 15-year-old boys and girls, including socio-economic environment, alcohol and drug use, sexual health, health habits, body image, and family and peer relationships [24]. Anonymity was maintained during data collection, and appropriate confidentiality measures were implemented. Comprehensive information on the HBSC sampling design and data collection methods is available in prior publications [25]. Briefly, HBSC applies a complex sampling design in which school classes serve as the primary sampling units and students are drawn to be representative by age and gender [25]. Countries implement single-stage cluster sampling with stratification by region and by grade, and classes are selected with probability proportional to size [26, 27]. The protocol recommends country-level targets of about 1,500 students per age group at 11, 13, and 15 years, with modest oversampling of 10% to 25% to accommodate non-response and age misalignment. Stratification is defined by age, geographic area, or school type and aims to cover at least 95% of the eligible population within the sampling frame; primary sampling units are selected systematically or randomly from all eligible schools, following a standardized protocol that specifies frames, selection procedures, instruments, and coding [25]. Prior to data collection, study procedures received ethical approval from the institutional ethics committee or other relevant board at the country or regional level and were endorsed by the World Health Organization to ensure compliance with ethical research standards. All countries participating in the study follow a standardized protocol. The protocol describes the methods for conducting the survey, the rules to be followed, and the coding procedures for the collected data [25, 28].

We conducted a retrospective secondary analysis of publicly accessible, de-identified data from the five most recent HBSC waves (2002, 2006, 2010, 2014, 2018). Missing data were handled by sequential exclusion, first removing records missing total breakfast days and then removing those missing psychometric complaints. Across different survey years, we first excluded participants with missing data of breakfast days (16,898 in 2002, 15,680 in 2006, 12,893 in 2010, 14,667 in 2014, and 20,527 in 2018), followed by those with missing psychometric complaints (4,675 in 2002, 6,890 in 2006, 7,603 in 2010, 15,694 in 2014, and 14,191 in 2018). The final sample analyzed in this study consisted of 918,564 adolescents across the five time points: 141,347 in 2002, 184,448 in 2006, 194,443 in 2010, 186,311 in 2014, and 212,015 in 2018. Supplementary Figure S1 provides the flowchart of participants.

### Measurement of breakfast frequency

Breakfast frequency was measured by the number of days per week. Participants were asked to indicate how many days they usually ate breakfast (defined as having more than a glass of milk or fruit juice) during the week and weekends, respectively. The categories of responses were: “I never have breakfast on weekdays”, “One day”, “Two days”, “Three days”, “Four days”, “Five days” for weekdays. In addition, for weekend: “I never eat breakfast on the weekend”, “I usually eat breakfast on only one day of the weekend”, “I usually eat breakfast on both Saturday and Sunday”. The responses were summed to derive total breakfast days per week, with possible scores ranging from 0 (breakfast skipping) to 7 (daily breakfast). This approach is supported by research about children’s and adolescents’ nutritional consumption [29, 30], which suggests that maintaining a consistent breakfast routine throughout the week is a reliable indicator of whether adolescents receive the continuous energy and nutritional support needed to meet the demands of their daily lives.

### Measurement of psychosomatic complaints

In this study, adolescents’ psychosomatic complaints were measured using an 8-item scale assessing the frequency of the following psychosomatic health complaints (feeling low, irritability or bad temper, feeling nervous, difficulty sleeping, dizziness, headache, stomachache, and backache) over the past 6 months. The response options for each question ranged from “daily”, “more than once a week”, “about every week”, “about every month”, to “rarely or never”. After reverse coding each item on a 0–4 scale, responses were aggregated to create a total score ranging from 0 to 32, where higher values indicated more frequent psychosomatic complaints. Cronbach’s alpha in our sample was 0.835, indicating that the items had a high internal consistency. The psychosomatic complaints score has demonstrated good validity and reliability in adolescent populations and has been widely applied in previous research [18, 31].

### Covariates

Covariate selection was informed by data availability and prior literature. Sociodemographic variables included age, sex, school grade, and socioeconomic status. Socioeconomic status was assessed using the Family Affluence Scale (FAS), a validated composite indicator widely employed in the HBSC study to reflect objective socioeconomic status [32]. The FAS consists of four items: family car ownership, bedroom occupancy, frequency of family holidays, and computer ownership. A total FAS score ranging from 0 to 9 was calculated by summing the responses to each item [32]. Body mass index (BMI) was derived from self-reported height and weight. The validity of self-reported data for estimating BMI in child and adolescent populations has been supported by previous research [33]. Physical activity was assessed through the item: “Over the past 7 days, on how many days were you physically active for a total of at least 60 minutes per day?”, with response options ranging from 0 to 7 days. Academic pressure was measured using a single item: “How pressured do you feel by the schoolwork you have to do?”, with responses ranging from “Not at all” to “A lot”. Experience of bullying was measured with the question: “How often have you been bullied at school in the past couple of months?”, with response options including “Never”, “Once or twice”, “2–3 times per month”, “Once a week”, and “Several times a week” [34]. A diet quality score was determined based on a combination of vegetables/salads, fruits, sweets, and sugar-sweetened beverages. Diet information at each survey in HBSC was assessed via a standardized food frequency questionnaire (FFQ) [35, 36].

### Statistical analysis

Summary statistics are presented stratified by different survey year. Categorical variables were presented as numbers and percentages, while continuous variables were presented as means and standard deviations. Baseline characteristics across different survey years were compared using ANOVA for continuous variables and Chi-square tests for categorical variables.

The primary estimand of this study is the descriptive cross-sectional association between the number of breakfast days per week and psychosomatic complaints scores within each survey wave, rather than the causal effect of changing breakfast behavior. A multilevel generalized additive model (GAM) was applied to estimate the association between breakfast consumption frequency and psychosomatic complaints, adjusted for age, gender, school grade, physical activity, BMI, FAS, academic pressure, the experience of being bullied, and survey year (only for overall participants). Among them, age and BMI were represented as smoothing splines. We employed penalized thin-plate regression splines (bs=“tp”) to represent smooth terms, with smoothing parameters selected via Restricted Maximum Likelihood (REML) to prevent over-fitting. The primary model was specified as: gam(Mental_Health_Score ~ s(total_breakfast_days, k = 8, bs=“tp”) + s(age, k = 6, bs=“tp”) + s(BMI, k = 6, bs=“tp”) + covariates + s(Country.number, bs=“re”) + s(Survey.year, bs=“re”)). Considering the multilevel structure of the data, where students are nested within schools, schools within countries, and countries within survey rounds, a multilevel framework was applied to account for clustering. In all analyses, the available HBSC sampling weights were normalized within country and survey year and applied in the models, with country and survey year treated as random effects (bs=“re”) to account for cross-national and temporal heterogeneity. Beyond adjusted mean differences (β), we additionally reported two standardized effect sizes: the percent of maximum possible (POMP) and the standardized mean difference (SMD), to enhance interpretability. We also present the estimated marginal means (adjusted mean scores) and their 95% CIs for each breakfast level of breakfast frequency, allowing for a direct comparison of the psychosomatic complaint burden across groups. To address multiple comparisons, we controlled the false discovery rate using the Benjamini-Hochberg False Discovery Rate (FDR) procedure (two-sided α = 0.05). Model fit was formally evaluated by comparing the penalized spline specification against linear and quadratic models using the Akaike Information Criterion (AIC) and Bayesian Information Criterion (BIC). The complexity of the smooth terms was evaluated using the Estimated Degrees of Freedom (EDF). Additionally, the first derivative of the smooth function and its 95% CI were calculated to identify ranges of significant marginal change and regions of statistical plateau (defined as the range where the 95% CI of the derivative included zero). Intraclass correlation coefficients (ICCs) were calculated to justify the multilevel framework. To assess cross-national consistency, we conducted a country-stratified meta-analysis and generated a forest plot with the I^2^ and τ^2^ statistics to evaluate the variability of associations across different settings.

The non-linear association of breakfast consumption frequency with psychosomatic complaints was examined through multilevel GAM analysis, with the same covariates. Subgroup analyses were performed to identify possible effect modifications of gender and school grade by adding an interaction term (breakfast consumption frequency × survey year, breakfast consumption frequency × survey year × gender, breakfast consumption frequency × survey year × school grade) to the model, and the modification was evaluated using *P*-interaction by likelihood ratio test.

To evaluate the robustness of our findings, we conducted four sets of sensitivity analyses. First, we restricted the analysis to participants with complete covariate data to assess the potential influence of missing data on the observed associations. Second, we further adjusted the models for parent-child communication variables (easy to talk with father and easy to talk with mother) [37]. Third, we restricted analyses to the two most recent waves (2014 and 2018) and additionally adjusted for family support to assess consistency under an expanded covariate set [38]. Fourth, to examine the possible inferences of participants with missing data (Supplementary Table S1), multiple imputation was used by fully conditional specification (FCS) to fill in missing values. Lastly, we conducted a five-fold cross-validation by randomly partitioning the dataset into five subsets and refitted the model on each fold to assess the stability of coefficient estimates.

All analyses were performed using R Statistical Software (version 4.5.0), utilizing the mgcv (version 1.9.4) and gratia (version 0.11.1) packages, with statistical significance set at a two-sided *P* value of < 0.05.

## Results

Table 1presents the descriptive statistics for adolescents across survey years. ໿We included 918,564 adolescents (mean age 13.59 ± 1.64 years), of whom 473,633 were girls (51.6%) across the five HBSC waves (2002–2018). Among them, 247,921 (32.9%) adolescents were in grade 5, the mean (SD) of BMI was 19.49 (3.50), and 41,189 (4.6%) adolescents reported no physical activity. In addition, 104,947 (11.6%) adolescents ໿felt a lot of academic pressure, and 616,492 (70.5%) adolescents reported no experience of bullying. Across survey years from 2002 to 2018, psychosomatic complaint scores gradually increased from a mean (SD) of 7.77 (6.14) in 2002 to 8.63 (6.72) in 2018 (*P* < 0.001), corresponding to an 11.1% relative increase. Over the same period, the proportion of breakfast skipping increased from 2.7% to 4.1% (*P* < 0.001), a 51.8% relative increase. Baseline comparability between included and excluded participants is summarized in Supplementary Table S2. The results indicated that the exposure showed negligible to small imbalance across all waves (SMD ≤ 0.129), while some lifestyle and psychosocial variables showed moderate imbalances in certain years.

**Table 1:** Baseline characteristics of study participants

Variable	Overall	2002	2006	2010	2014	2018	*P*
Total participants	918,564	141,347	184,448	194,443	186,311	212,015	
Age	13.59 (1.64)	13.57 (1.66)	13.63 (1.64)	13.59 (1.64)	13.61 (1.63)	13.54 (1.63)	< 0.001
Gender							
Male	444,931 (48.4)	68,071 (48.2)	89,211 (48.4)	94,329 (48.5)	90,390 (48.5)	102,930 (48.5)	0.095
Female	473,633 (51.6)	73,276 (51.8)	95,237 (51.6)	100,114 (51.5)	95,921 (51.5)	109,085 (51.5)	
School grade							
Grade 5	247,921 (32.9)	47,304 (33.5)	58,513 (31.9)	48,425 (33.6)	41,378 (32.3)	52,301 (33.4)	< 0.001
Grade 7	257,256 (34.1)	48,521 (34.3)	62,850 (34.3)	47,829 (33.2)	43,968 (34.4)	54,088 (34.5)	
Grade 9	248,487 (33.0)	45,522 (32.2)	62,097 (33.8)	47,837 (33.2)	42,615 (33.3)	50,416 (32.2)	
Physical activity							
No Physical activity	41,189 (4.6)	6146 (4.6)	8498 (4.7)	8932 (4.7)	7386 (4.0)	10,227 (4.9)	< 0.001
1 days	67,958 (7.6)	12,291 (9.2)	13,847 (7.7)	14,418 (7.5)	12,471 (6.8)	14,931 (7.2)	
2 days	120,389 (13.4)	21,017 (15.6)	24,332 (13.5)	25,906 (13.6)	23,317 (12.7)	25,817 (12.4)	
3 days	149,810 (16.7)	23,988 (17.9)	29,660 (16.4)	31,757 (16.6)	29,975 (16.4)	34,430 (16.5)	
4 days	136,790 (15.2)	19,769 (14.7)	26,772 (14.8)	29,687 (15.5)	27,888 (15.2)	32,674 (15.7)	
5 days	129,531 (14.4)	17,418 (13.0)	25,375 (14.1)	27,545 (14.4)	27,566 (15.1)	31,627 (15.2)	
6 days	83,612 (9.3)	10,766 (8.0)	16,070 (8.9)	18,149 (9.5)	18,202 (9.9)	20,425 (9.8)	
7 days	168,078 (18.7)	22,932 (17.1)	36,002 (19.9)	34,732 (18.2)	36,229 (19.8)	38,183 (18.3)	
BMI	19.49 (3.50)	19.19 (3.30)	19.43 (3.39)	19.62 (3.52)	19.58 (3.58)	19.55 (3.64)	< 0.001
Family Affluence Scale							
0	8769 (1.0)	2116 (1.5)	2485 (1.4)	1244 (0.7)	1159 (0.7)	1765 (0.9)	< 0.001
1	22,908 (2.6)	5523 (4.0)	5451 (3.0)	3370 (1.8)	3655 (2.1)	4909 (2.4)	
2	45,341 (5.1)	10,239 (7.4)	10,343 (5.8)	7275 (3.9)	7895 (4.5)	9589 (4.7)	
3	77,555 (8.8)	16,705 (12.1)	18,086 (10.1)	13,596 (7.3)	13,848 (8.0)	15,320 (7.5)	
4	115,996 (13.1)	24,140 (17.4)	26,815 (15.0)	22,109 (11.8)	20,644 (11.9)	22,288 (10.9)	
5	147,134 (16.7)	26,502 (19.1)	32,059 (17.9)	30,020 (16.1)	28,109 (16.1)	30,444 (14.9)	
6	163,204 (18.5)	24,081 (17.4)	32,191 (18.0)	35,653 (19.1)	34,027 (19.5)	37,252 (18.2)	
7	140,248 (15.9)	17,070 (12.3)	26,008 (14.5)	33,165 (17.7)	29,342 (16.9)	34,663 (17.0)	
8	97,926 (11.1)	8481 (6.1)	16,460 (9.2)	24,787 (13.3)	21,027 (12.1)	27,171 (13.3)	
9	64,043 (7.3)	3565 (2.6)	9260 (5.2)	15,784 (8.4)	14,348 (8.2)	21,086 (10.3)	
Academic pressure							
Not at all	185,115 (20.4)	28,120 (20.1)	35,165 (19.3)	39,482 (20.5)	39,417 (21.5)	42,931 (20.5)	< 0.001
A little	398,606 (44.0)	62,386 (44.6)	80,713 (44.4)	85,830 (44.7)	80,942 (44.2)	88,735 (42.4)	
Some	217,380 (24.0)	34,326 (24.6)	46,217 (25.4)	45,927 (23.9)	41,535 (22.7)	49,375 (23.6)	
A lot	104,947 (11.6)	14,953 (10.7)	19,888 (10.9)	20,896 (10.9)	21,176 (11.6)	28,034 (13.4)	
Experienced bullying							
Never	616,492 (70.5)	93,568 (66.8)	118,831 (68.2)	132,182 (71.1)	126,826 (72.2)	145,085 (72.9)	< 0.001
Once or twice	158,947 (18.2)	28,954 (20.7)	33,915 (19.5)	33,266 (17.9)	30,292 (17.3)	32,520 (16.3)	
2–3 times per month	39,280 (4.5)	6494 (4.6)	8340 (4.8)	8271 (4.5)	7531 (4.3)	8644 (4.3)	
Once/week	25,472 (2.9)	4358 (3.1)	5608 (3.2)	5266 (2.8)	4785 (2.7)	5455 (2.7)	
Several times/week	34,534 (3.9)	6800 (4.9)	7428 (4.3)	6876 (3.7)	6142 (3.5)	7288 (3.7)	
Diet quality score							< 0.001
Poor	496,403 (54.4)	89,349 (63.5)	108,996 (59.5)	104,524 (54.1)	93,339 (50.4)	100,195 (47.5)	
Intermediate	269,647 (29.5)	36,544 (26.0)	49,887 (27.2)	57,578 (29.8)	58,042 (31.4)	67,596 (32.1)	
Ideal	146,871 (16.1)	14,816 (10.5)	24,241 (13.2)	31,033 (16.1)	33,747 (18.2)	43,034 (20.4)	
Psychosomatic complaints score	8.19 (6.55)	7.77 (6.14)	8.05 (6.42)	8.13 (6.52)	8.19 (6.80)	8.63 (6.72)	< 0.001
Breakfast days							
0 days	29,525 (3.2)	3772 (2.7)	5329 (2.9)	5673 (2.9)	5982 (3.2)	8769 (4.1)	< 0.001
1 days	39,460 (4.3)	5674 (4.0)	7502 (4.1)	8203 (4.2)	7428 (4.0)	10,653 (5.0)	
2 days	102,115 (11.1)	15,293 (10.8)	21,275 (11.5)	21,318 (11.0)	19,994 (10.7)	24,235 (11.4)	
3 days	40,159 (4.4)	5506 (3.9)	7743 (4.2)	8563 (4.4)	7706 (4.1)	10,641 (5.0)	
4 days	47,015 (5.1)	6356 (4.5)	9202 (5.0)	10,127 (5.2)	9331 (5.0)	11,999 (5.7)	
5 days	72,534 (7.9)	9915 (7.0)	14,409 (7.8)	15,963 (8.2)	14,643 (7.9)	17,604 (8.3)	
6 days	83,356 (9.1)	11,927 (8.4)	16,147 (8.8)	18,307 (9.4)	17,058 (9.2)	19,917 (9.4)	
7 days	504,400 (54.9)	82,904 (58.7)	102,841 (55.8)	106,289 (54.7)	104,169 (55.9)	108,197 (51.0)	

*Abbreviations*: *BMI*, body mass index

Data presented as means (standard deviation) for continuous variables and numbers (percentages) for categorical variables. Examining differences among different survey years based on *t* test (for continuous variables) and *χ*^*2*^ test (for categorical variables)

Associations of Total Breakfast Days Categories with psychosomatic complaints

Table 2 shows the associations between breakfast consumption frequency and psychosomatic complaints among overall adolescents and across different survey years. Lower breakfast consumption frequency was consistently associated with a higher psychosomatic complaints score (*P* for trend < 0.001). Among the overall sample, adolescents who skipped breakfast (0 days) reported significantly higher symptom burden compared to those who consumed breakfast daily (7 days), with an adjusted mean score of 10.17 (95% CI: 10.08–10.26) versus 7.41 (95% CI: 7.39–7.43), respectively. The fully adjusted β for breakfast skipping was 2.84 (95% CI: 2.75–2.93), which corresponds to a POMP of 8.9% (95% CI: 8.6–9.2) on the 0–32 scale and a SMD of 0.49 (95% CI: 0.47–0.50). This association was robust across all survey waves. In 2002, breakfast skipping was associated with a significantly higher psychosomatic complaints score (β: 2.53, 95% CI: 2.32–2.74; *P* < 0.001), corresponding to an SMD of 0.46. While the magnitude of this association observed in 2018 reached an adjusted β of 2.99 (95% CI: 2.80–3.18) and an SMD of 0.50, the overall association remained consistent over time (Table 2). After controlling the FDR using the Benjamini-Hochberg procedure, all reported associations across all categories and survey years remained statistically significant (*P*-FDR < 0.001) (Table 2).

**Table 2:** Associations of Total Breakfast Days Categories with psychosomatic complaints

Breakfast Frequency (Days/Week)	β (95% CI)	Adjusted Mean Score (95% CI)	POMP (95% CI)	SMD (95% CI)	*P*-FDR
Overall					
0 days	2.84 (2.75–2.93)	10.17 (10.08–10.26)	8.9 (8.6–9.2)	0.49 (0.47–0.50)	< 0.001
1 days	2.61 (2.53–2.69)	9.88 (9.81–9.96)	8.2 (7.9–8.4)	0.45 (0.43–0.46)	< 0.001
2 days	1.51 (1.46–1.56)	8.93 (8.88–8.97)	4.7 (4.6–4.9)	0.26 (0.25–0.27)	< 0.001
3 days	1.57 (1.49–1.65)	8.96 (8.89–9.03)	4.9 (4.7–5.1)	0.27 (0.26–0.28)	< 0.001
4 days	1.45 (1.37–1.52)	8.80 (8.74–8.87)	4.5 (4.3–4.7)	0.25 (0.24–0.26)	< 0.001
5 days	1.20 (1.15–1.26)	8.54 (8.49–8.59)	3.8 (3.6–3.9)	0.21 (0.20–0.22)	< 0.001
6 days	0.92 (0.87–0.97)	8.25 (8.20–8.30)	2.9 (2.7-3.0)	0.16 (0.15–0.17)	< 0.001
7 days	Ref.	7.41 (7.39–7.43)	Ref.	Ref.	Ref.
* P*-trend	< 0.001				
Survey 2002					
0 days	2.53 (2.32–2.74)	9.71 (9.50–9.93)	7.9 (7.2–8.6)	0.46 (0.42–0.49)	< 0.001
1 days	2.26 (2.09–2.43)	9.40 (9.22–9.57)	7.1 (6.5–7.6)	0.41 (0.38–0.44)	< 0.001
2 days	1.36 (1.25–1.47)	8.58 (8.47–8.68)	4.3 (3.9–4.6)	0.25 (0.23–0.26)	< 0.001
3 days	1.35 (1.18–1.52)	8.55 (8.38–8.72)	4.2 (3.7–4.8)	0.24 (0.21–0.27)	< 0.001
4 days	1.21 (1.05–1.37)	8.35 (8.20–8.51)	3.8 (3.3–4.3)	0.22 (0.19–0.25)	< 0.001
5 days	1.06 (0.93–1.19)	8.24 (8.11–8.36)	3.3 (2.9–3.7)	0.19 (0.17–0.21)	< 0.001
6 days	0.76 (0.64–0.88)	7.95 (7.83–8.06)	2.4 (2.0-2.7)	0.14 (0.11–0.16)	< 0.001
7 days	Ref.	7.22 (7.17–7.26)	Ref.	Ref.	Ref.
* P*-trend	< 0.001				
Survey 2006					
0 days	2.75 (2.56–2.94)	9.93 (9.75–10.12)	8.6 (8.0-9.2)	0.48 (0.45–0.52)	< 0.001
1 days	2.38 (2.22–2.54)	9.64 (9.48–9.79)	7.4 (6.9–7.9)	0.42 (0.39–0.45)	< 0.001
2 days	1.48 (1.38–1.58)	8.78 (8.69–8.87)	4.6 (4.3–4.9)	0.26 (0.24–0.28)	< 0.001
3 days	1.60 (1.44–1.75)	8.82 (8.67–8.97)	5.0 (4.5–5.5)	0.28 (0.25–0.31)	< 0.001
4 days	1.44 (1.30–1.58)	8.68 (8.54–8.81)	4.5 (4.1-5.0)	0.25 (0.23–0.28)	< 0.001
5 days	1.30 (1.19–1.42)	8.54 (8.43–8.64)	4.1 (3.7–4.4)	0.23 (0.21–0.25)	< 0.001
6 days	0.92 (0.81–1.02)	8.14 (8.04–8.24)	2.9 (2.5–3.2)	0.16 (0.14–0.18)	< 0.001
7 days	Ref.	7.34 (7.30–7.38)	Ref.	Ref.	Ref.
* P*-trend	< 0.001				
Survey 2010					
0 days	2.59 (2.38–2.81)	9.82 (9.61–10.02)	8.1 (7.4–8.8)	0.45 (0.41–0.48)	< 0.001
1 days	2.53 (2.35–2.71)	9.76 (9.59–9.93)	7.9 (7.4–8.5)	0.44 (0.41–0.47)	< 0.001
2 days	1.43 (1.32–1.55)	8.92 (8.81–9.02)	4.5 (4.1–4.8)	0.25 (0.23–0.27)	< 0.001
3 days	1.70 (1.52–1.87)	9.07 (8.91–9.24)	5.3 (4.8–5.9)	0.29 (0.26–0.32)	< 0.001
4 days	1.36 (1.19–1.52)	8.80 (8.64–8.95)	4.2 (3.7–4.7)	0.23 (0.21–0.26)	< 0.001
5 days	1.11 (0.98–1.24)	8.46 (8.34–8.58)	3.5 (3.1–3.9)	0.19 (0.17–0.21)	< 0.001
6 days	1.07 (0.95–1.19)	8.36 (8.25–8.47)	3.3 (3.0-3.7)	0.18 (0.16–0.20)	< 0.001
7 days	Ref.	7.40 (7.35–7.44)	Ref.	Ref.	Ref.
* P*-trend	< 0.001				
Survey 2014					
0 days	2.92 (2.69–3.15)	10.32 (10.10-10.53)	9.1 (8.4–9.8)	0.49 (0.45–0.52)	< 0.001
1 days	2.63 (2.42–2.83)	9.96 (9.77–10.16)	8.2 (7.6–8.8)	0.44 (0.40–0.47)	< 0.001
2 days	1.49 (1.36–1.62)	8.98 (8.86–9.10)	4.7 (4.3–5.1)	0.25 (0.23–0.27)	< 0.001
3 days	1.62 (1.43–1.82)	9.01 (8.82–9.20)	5.1 (4.5–5.7)	0.27 (0.24–0.30)	< 0.001
4 days	1.64 (1.46–1.82)	9.02 (8.86–9.19)	5.1 (4.6–5.7)	0.27 (0.24–0.30)	< 0.001
5 days	1.24 (1.10–1.39)	8.59 (8.45–8.72)	3.9 (3.4–4.3)	0.21 (0.18–0.23)	< 0.001
6 days	0.93 (0.80–1.07)	8.27 (8.14–8.39)	2.9 (2.5–3.3)	0.16 (0.13–0.18)	< 0.001
7 days	Ref.	7.39 (7.34–7.44)	Ref.	Ref.	Ref.
* P*-trend	< 0.001				
Survey 2018					
0 days	2.99 (2.80–3.18)	10.80 (10.63–10.98)	9.3 (8.8–9.9)	0.50 (0.47–0.53)	< 0.001
1 days	2.95 (2.78–3.11)	10.54 (10.39–10.70)	9.2 (8.7–9.7)	0.49 (0.46–0.52)	< 0.001
2 days	1.61 (1.50–1.72)	9.32 (9.22–9.42)	5.0 (4.7–5.4)	0.27 (0.25–0.29)	< 0.001
3 days	1.40 (1.24–1.55)	9.27 (9.13–9.42)	4.4 (3.9–4.9)	0.23 (0.21–0.26)	< 0.001
4 days	1.41 (1.26–1.56)	9.18 (9.04–9.32)	4.4 (3.9–4.9)	0.23 (0.21–0.26)	< 0.001
5 days	1.15 (1.02–1.27)	8.86 (8.75–8.98)	3.6 (3.2-4.0)	0.19 (0.17–0.21)	< 0.001
6 days	0.83 (0.71–0.95)	8.53 (8.42–8.64)	2.6 (2.2-3.0)	0.14 (0.12–0.16)	< 0.001
7 days	Ref.	7.70 (7.65–7.75)	Ref.	Ref.	Ref.
* P*-trend	< 0.001				

The βs and 95% CIs were extracted from multilevel generalized additive model (GAM), with psychosomatic complaints score as the outcome and variable of total breakfast days categories as the exposure. POMP was computed as β divided by the 0-32 outcome range; SMD was computed as β divided by the (weighted) SD of the outcome, with 95% confidence intervals (CIs) obtained by scaling the β confidence limits by the same SD

The *P* value for FDR were extracted from Benjamini-Hochberg False Discovery Rate (FDR) analysis, with psychosomatic complaints as the outcome and variable of total breakfast days categories as the exposure. *P* value for trend calculated treating the total breakfast days as a continuous variable

The 7 days category (daily breakfast) was used as the reference

Models were adjusted for survey year (only for overall participants), gender, school grade, physical activity, Family Affluence Scale, academic pressure, having experienced bullying, diet quality score, and smooth term of age and BMI

*Abbreviations*: *CI*, confidence interval; *POMP*, Percent of Maximum Possible; *SMD*, the standardized mean difference; *FDR*, False Discovery Rate; *Ref*, reference

**Fig. 1:**
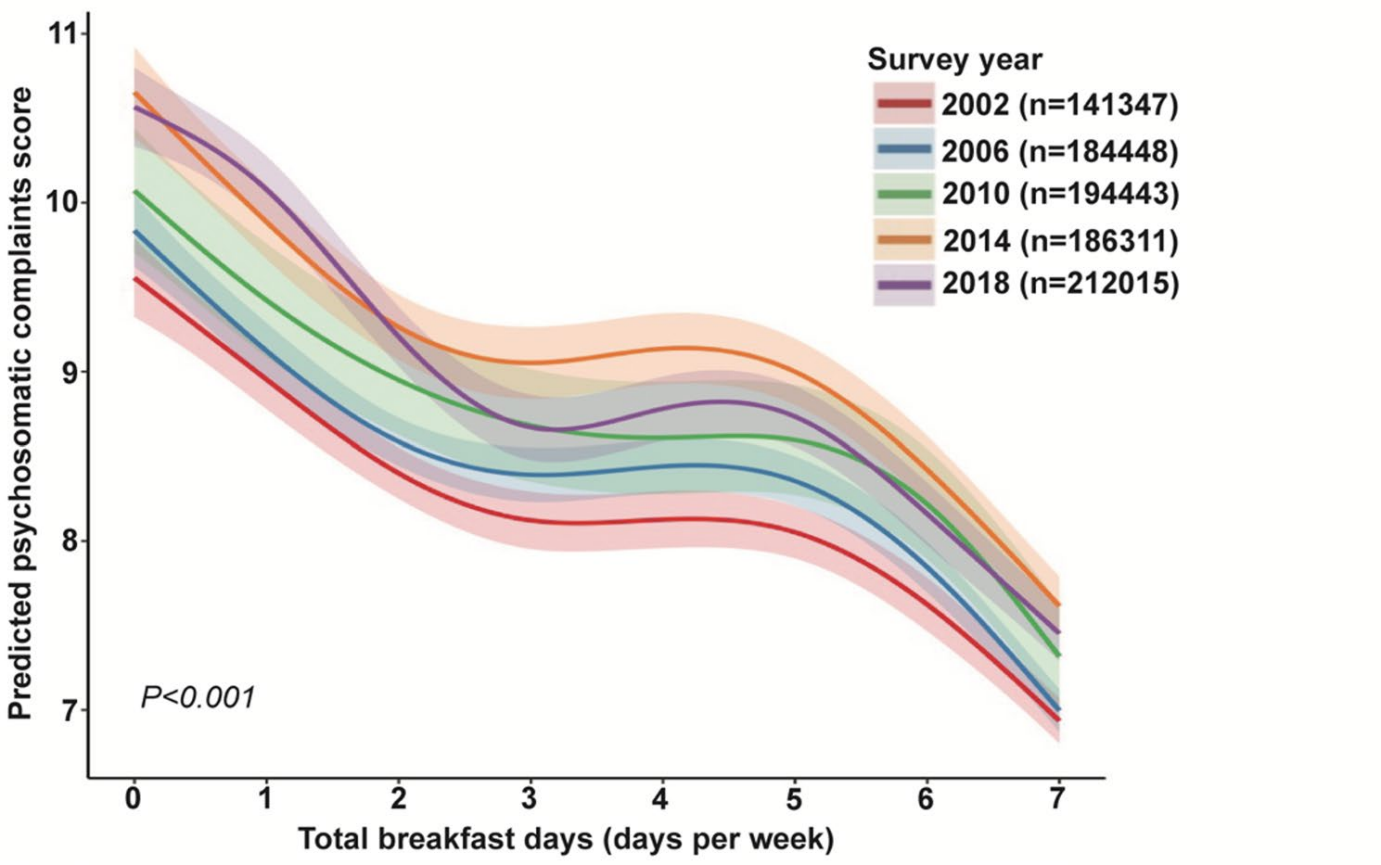
Adjusted association between breakfast frequency and psychosomatic complaints score by survey year. The figure shows the smoothed, non-linear association between breakfast frequency (total breakfast days per week) and psychosomatic complaints, estimated from multilevel GAMs. Curves represent adjusted model-predicted psychosomatic complaints, and shaded areas indicate 95% confidence intervals. Models were adjusted for gender, school grade, physical activity, Family Affluence Scale, academic pressure, having experienced bullying, diet quality score, and smooth terms for age and BMI. Psychosomatic complaints were assessed using an 8-item scale including feeling low, irritability, nervousness, sleep difficulties, dizziness, headache, stomachache, and backache. The *P* value corresponds to the test of nonlinearity for breakfast frequency in the multilevel GAM

Figure 1illustrates the non-linear associations between breakfast frequency and psychosomatic complaints across different survey years (*P* for nonlinear < 0.001). The curves show a stepped pattern, with a marked decline in psychosomatic complaint scores as breakfast frequency increases from 0 to 3 days per week, followed by diminishing marginal differences between 3 and 5 days, and a further reduction approaching daily consumption. Overall, the inverse non-linear association was consistent across survey years (*P* < 0.001). Model comparison confirmed that the penalized spline GAM provided a significantly better fit than both linear (ΔAIC = 1684.1 and ΔBIC = 1617.0) and quadratic models (ΔAIC = 1406.6 and ΔBIC = 1350.7), supporting a non-linear association (Supplementary Table S3). Furthermore, analysis of the first derivative of the smooth function (EDF = 6.87) (Supplementary Table S4) identified a range starting at approximately 4.8 days/week, statistically confirming a plateau-like flattening of the association where the 95% confidence interval of the derivative included zero (Supplementary Figure S2). The multilevel framework was statistically justified, although the country-level ICC was < 0.001 after adjusting for the full set of covariates. In the country-stratified meta-analysis (Supplementary Figure S3), the inverse association between breakfast frequency and psychosomatic complaints was reproducible across 100% of the 46 included countries and regions. While significant heterogeneity in effect magnitude was observed (I^2^ = 96.1%, τ^2^ = 0.0159, *P* < 0.001), the universal directional consistency (all 46 country-specific beta coefficients were negative) demonstrates that the global trend is robust and not driven by any specific region. 

**Fig. 2:**
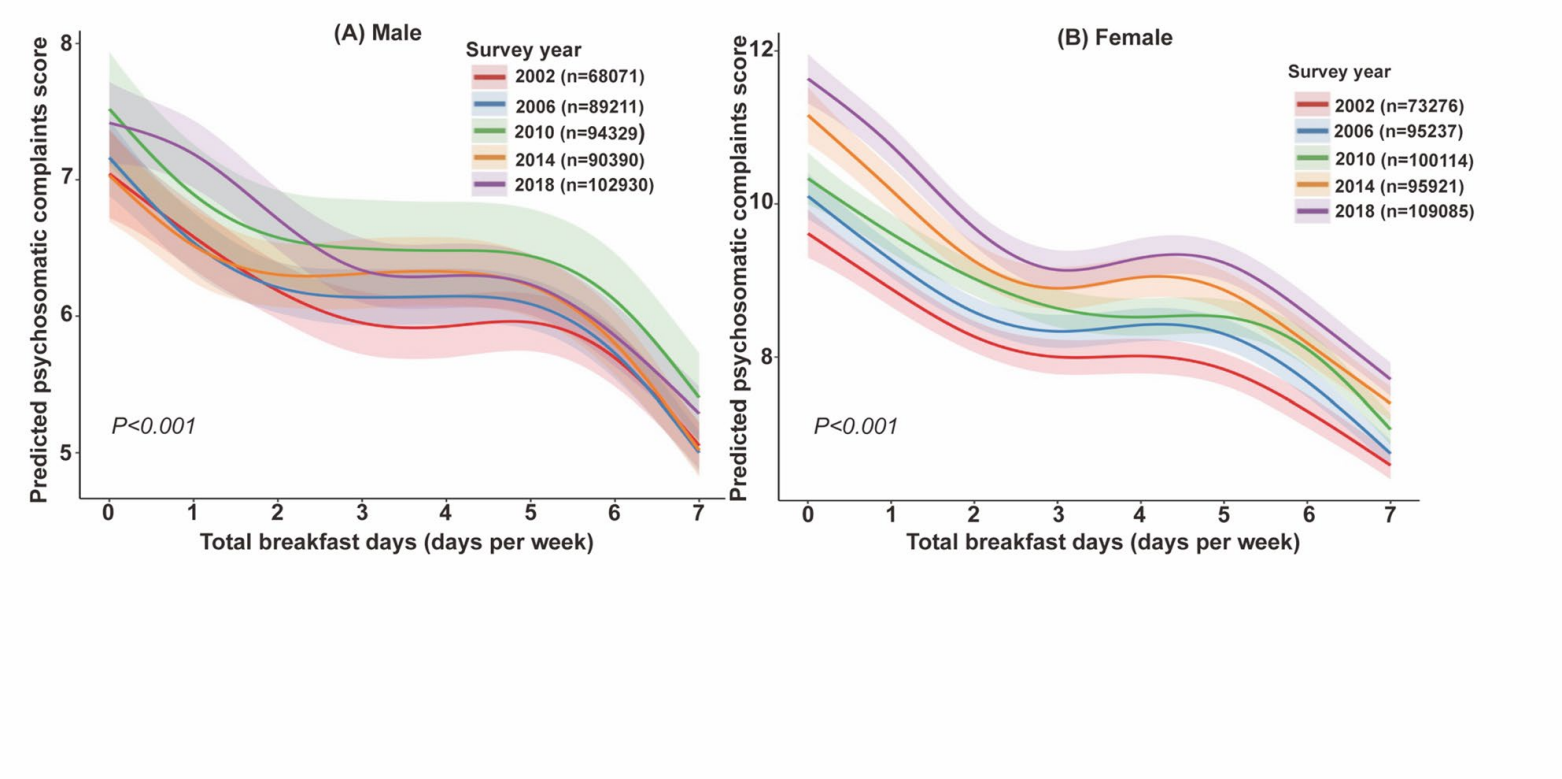
Adjusted association between breakfast frequency and psychosomatic complaints score by survey year and sex. The figure shows the smoothed, non-linear association between breakfast frequency (total breakfast days per week) and psychosomatic complaints, estimated from multilevel GAMs. Curves represent adjusted model-predicted psychosomatic complaints, and shaded areas indicate 95% confidence intervals. Models were adjusted for gender, school grade, physical activity, Family Affluence Scale, academic pressure, having experienced bullying, diet quality score, and smooth terms for age and BMI. Psychosomatic complaints were assessed using an 8-item scale including feeling low, irritability, nervousness, sleep difficulties, dizziness, headache, stomachache, and backache. The *P* value corresponds to the test of nonlinearity for breakfast frequency in the multilevel GAM

The association between breakfast consumption and psychosomatic complaints stratified by gender was shown in Supplementary Table S5. Gender-stratified multilevel GAMs showed an inverse association between breakfast consumption frequency and psychosomatic complaints score in both boys and girls (*P* for trend < 0.001 in each subgroup). The association of breakfast consumption frequency with psychosomatic complaints was stronger in girls than boys (*P* for interaction < 0.001). For example, compared with daily breakfast (7 days), breakfast skipping was associated with a β of 3.39 (95% CI 3.26–3.52) in girls and 2.02 (95% CI 1.90–2.15) in boys, indicating effect modification by gender. Figure 2 presents the gender-stratified non-linear associations between breakfast frequency and psychosomatic complaints. In both boys and girls, higher breakfast frequency was associated with lower psychosomatic complaint score. Gender-stratified GAMs in Fig. 2 show a similar inverse non-linear association across sexes (*P* for nonlinearity < 0.001), while girls (Panel B) have higher psychosomatic complaint scores than boys (Panel A) at every level of breakfast frequency.

**Fig. 3:**
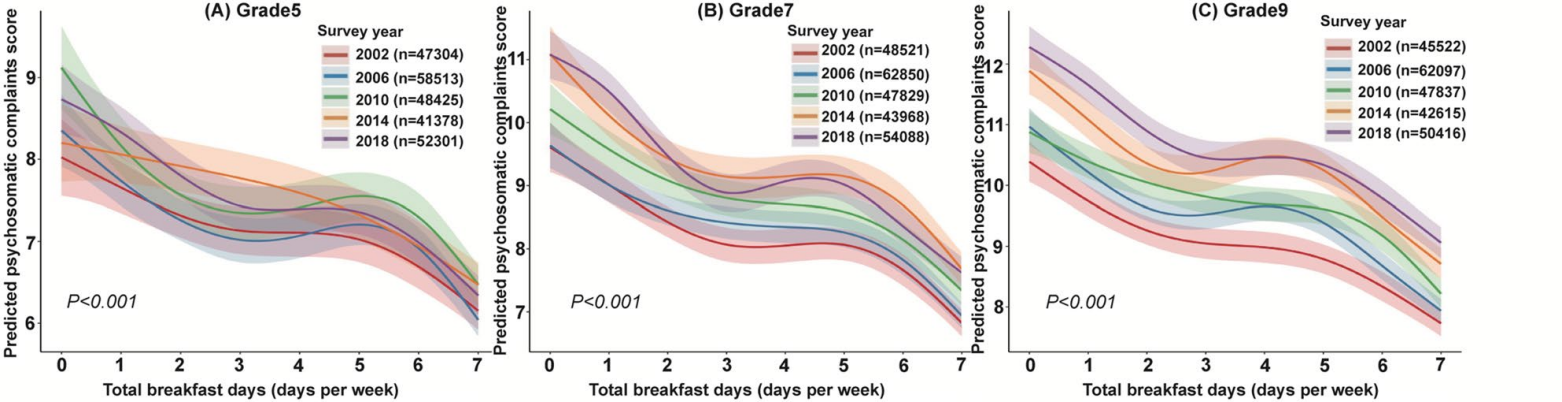
Adjusted association between breakfast frequency and psychosomatic complaints score by survey year and school grade.The figure shows the smoothed, non-linear association between breakfast frequency (total breakfast days per week) and psychosomatic complaints, estimated from multilevel GAMs. Curves represent adjusted model-predicted psychosomatic complaints, and shaded areas indicate 95% confidence intervals. Models were adjusted for gender, school grade, physical activity, Family Affluence Scale, academic pressure, having experienced bullying, diet quality score, and smooth terms for age and BMI. Psychosomatic complaints were assessed using an 8-item scale including feeling low, irritability, nervousness, sleep difficulties, dizziness, headache, stomachache, and backache. The *P* value corresponds to the test of nonlinearity for breakfast frequency in the multilevel GAM

The association between breakfast consumption and psychosomatic complaints stratified by school grade was shown in Supplementary Table S6. Grade-stratified multilevel GAMs showed that higher breakfast frequency was associated with lower psychosomatic complaints score among different school grade subgroups (*P* for trend < 0.001 in each subgroup). Associations between breakfast frequency and psychosomatic complaints were stronger in higher school grades than in lower grades, with adjusted β values of 2.07 (95% CI, 1.86–2.28) in grade 5, 2.88 (95% CI, 2.75–3.01) in grade 7, and 2.98 (95% CI, 2.82–3.14) in grade 9, indicating a significant effect modification by grade (*P* for interaction < 0.001). Figure 3 shows the grade-stratified non-linear associations of breakfast consumption with psychosomatic complaints. Among different school grades, higher breakfast frequency was associated with lower psychosomatic complaint score. A non-linear association was observed across all grades (*P* for nonlinearity < 0.001). At any given level of breakfast frequency, psychosomatic complaint scores increased with grade, with the highest scores in grade 9 and the lowest in grade 5. Formal interaction tests revealed that while the association between breakfast frequency and psychosomatic complaints significantly varied by gender and school grade (both ​*P* for interaction < 0.001), the inverse dose-response pattern and the characteristic non-linear plateau remained remarkably consistent across all demographic subgroups (Supplementary Figure S4).

We conducted several sensitivity analyses to examine the robustness of findings and presented the additional details in Supplementary Table S7-S11. First, restricting the sample to participants with complete covariate information yielded estimates that were virtually identical to the main analysis (Supplementary Table S7). Second, further adjustment for parent-child communication (easy to talk with father and mother) produced attenuated but still substantial associations (Supplementary Table S8). Third, limiting the analysis to the two most recent waves (2014 and 2018) and additionally adjusting for family support replicated the similar associations between breakfast frequency and psychosomatic complaints (Supplementary Table S9). Furthermore, in a sensitivity analysis using multiple imputation with fully conditional specification, the estimates pooled across imputations were highly consistent with the primary results (Supplementary Table S10). Finally, the same pattern of results was observed in the sensitivity analysis using 5-fold cross-validation (Supplementary Table S11).

## Discussion

Utilizing repeated cross-sectional data from over 900,000 adolescents surveyed between 2002 and 2018 across 45 countries, this study examined the non-linear association between breakfast frequency and psychosomatic complaints. Overall, breakfast frequency showed an inverse, non-linear association with psychosomatic complaints, and this association was similar across survey years, gender, and grade. At any given level of breakfast frequency, females reported higher psychosomatic complaint scores than males, and scores were higher among adolescents in higher grades.

Previous studies have reported that breakfast skipping is associated with poorer mental health and psychosomatic complaints [7, 8, 23]. For example, psychiatric distress was more common among breakfast skippers in Iranian children and adolescents [8] and a cross-sectional study of Greek adolescents aged 11–15 years found that fewer breakfast days per week were associated with worse psychological complaints [23]. Using pooled HBSC data spanning six survey waves across 45 countries, our study extends this literature by providing harmonized, multi-country estimates over time. We observed an inverse, non-linear association between breakfast frequency (days per week) and psychosomatic complaints, consistent with prior work suggesting that fewer breakfast days are associated with higher self-reported psychological complaints. Beyond supporting this overall direction, our findings further indicate that the shape of the association is non-linear and broadly similar across survey rounds, with comparable direction and magnitude. The GAM curves suggest that psychosomatic complaints decrease most steeply when moving from 0 to about 3 days per week, show smaller differences between roughly 3 and 5 days, and decrease further as intake approaches daily. This curve shape aligns with frequency categories commonly used in a prior study(e.g., 0–2 days, 3–5 days, and daily) [30], supporting the interpretability of the non-linear association.

In terms of magnitude, compared with adolescents reporting daily breakfast, those reporting no breakfast days had an adjusted difference of 2.84 points on the 0–32 psychosomatic complaints scale. This difference corresponds to 8.9% of POMP and a SMD of 0.49, indicating a small-to-moderate elevation in symptom burden. At the item level, this translates to an average shift of approximately 0.38 response categories per symptom across all eight items, suggesting a modest increase in the frequency of common complaints rather than a change in a single domain. Although the psychosomatic complaints score is a non-diagnostic, self-reported scale and does not map directly onto individual clinical thresholds, these standardized differences may still be meaningful at the population level. Overall, in a context where breakfast skipping is becoming more common and adolescent mental health concerns are increasingly salient. Our findings suggest that for adolescents who cannot eat breakfast every day due to social, family, or personal constraints, maintaining breakfast on several days per week (at least three) was associated with fewer psychosomatic complaints than skipping breakfast entirely.

Regarding the magnitude of the observed association, the SMD of approximately 0.49 between breakfast skippers and daily consumers represents a moderate effect size (Cohen’s d ≈ 0.5). Similarly, a PPOMP score difference of 8.9% suggests a discernable but not necessarily acute shift in symptom burden at the individual level. It should be explicitly noted that no formal MCID has been established to date for the 8-item HBSC psychosomatic complaints scale. Consequently, while these differences may appear moderate for a single individual, their implications at the population level are substantial, especially given our extensive sample size. From a public health perspective, even minor shifts in the population mean of a symptom distribution can lead to a significant increase in the cumulative burden of psychosomatic complaints across the adolescent population. Therefore, our findings should be interpreted as identifying infrequent breakfast consumption as a robust non-causal marker for high-risk groups, providing a strategic benchmark for school-based health screenings rather than a direct clinical diagnostic threshold.

Furthermore, our study identified that female and adolescents in higher grades had higher psychosomatic complaint scores. Previous studies showed stronger associations between breakfast skipping and unhealthy behaviors in girls than in boys [39]. Although the current data did not demonstrate a significant sex difference in this association, a gender-specific effect was anticipated given that girls are more likely to engage in dieting behaviors, and female dieters are three times more likely to skip breakfast than non-dieters. Furthermore, the tendency among girls to simultaneously restrict food intake and increase physical activity for weight management may further complicate the interplay between these behaviors [40]. Breakfast skipping has been linked to irregular eating patterns and unhealthy weight control behaviors (such as restrictive dieting), particularly among adolescents with higher school grades, which may lead to psychological stress and mood instability [41, 42].

The main advantages of this study include a large, nationally representative sample of more than 900,000 adolescents from multiple countries, which provides sufficient statistical power and enhances the generalizability of the results. In addition, stratified analyses by sex and grade level enabled the identification of subgroup-specific associations, which provided a useful basis for informing future research and intervention efforts. The present study also has some limitations. First, although multiple survey rounds were included, this remains a repeated cross-sectional study. Although we analyzed multiple survey rounds spanning 16 years, these data represent independent cohorts of adolescents rather than longitudinal follow-ups of the same individuals. Consequently, we cannot assess within-person changes or determine the temporal order of the observed associations. The differences in association estimates across survey years should not be interpreted as a temporal causal process or an evolving biological effect. Instead, these variations may reflect evolving reporting practices regarding psychosomatic complaints over the past two decades. Second, the breakfast measure in this study reflects frequency and regularity rather than nutritional quality. The HBSC definition does not capture energy content or composition, which may lead to residual confounding by diet quality. However, this standardized threshold enables consistent characterization of non-linear association across a vast, multi-country sample where detailed dietary records were unavailable. To mitigate this, we adjusted for a composite diet quality score to account for broader eating patterns. Third, another potential limitation is reporting bias due to the use of self-reported questionnaires. For example, in the present study, we used an eight-item questionnaire to construct psychosomatic complaints score as a non-diagnostic, symptom-based proxy outcome, rather than a validated psychiatric endpoint or positive well-being measure. Interpretations of self-reported items may differ across countries and cultures, which could introduce reporting heterogeneity and measurement error and, in turn, affect the comparability of scores across settings. Fourth, unmeasured or residual confounding cannot be excluded. Specifically, the HBSC pooled dataset lacks harmonized data on sleep duration and chronotype, which are linked to both breakfast habits and symptom reporting. Although our outcome measure (psychosomatic complaints) captures sleep-related distress, we cannot account for individual circadian preferences. Similarly, while sensitivity analyses using parental communication proxies suggests robust results, the absence of detailed family structure and breakfast-specific parental rules remains a limitation. Fifth, because complete-case participants differed in some respects from those excluded, selection bias cannot be ruled out. Finally, although we conducted an internal validation using 5-fold cross-validation, the majority of participants came from European and North American countries, and the lack of external validation in other populations means this may limit the applicability of the results to predominantly low-income populations. Conducting country-specific analyses represents an important direction for future research.

## Conclusions

In summary, based on this HBSC study of more than 900,000 adolescents, we found that breakfast frequency showed an inverse, non-linear association with psychosomatic complaints. This association was broadly consistent across survey rounds. In addition, this association was more pronounced among females and adolescents in higher school grades. These findings may offer valuable guidance for public health and school-based interventions, emphasizing the importance of promoting regular breakfast consumption and fostering healthy dietary habits for adolescents’ mental health.

.

## Supplementary Information

Supplementary Material 1.

## Data Availability

The data are publicly available and can be accessed here (https://hbsc.org/data/) **.**.
