# Supplementary material for: Breakfast frequency and psychosomatic complaints among adolescents: a repeated cross-sectional analysis of the HBSC study
Source: Arch Public Health. 2026 Apr 10;84:114. doi: 10.1186/s13690-026-01914-2 (PMC13181872; doi:10.1186/s13690-026-01914-2)
Supplement: Supplementary file 1 — Supplementary Material 1. [file 13690_2026_1914_MOESM1_ESM.docx]

**Supplementary Online Contents**

Breakfast Frequency and Psychosomatic Complaints among Adolescents: a Repeated Cross-Sectional Analysis of the HBSC Study

Yuzhong Duan, Jiao Yang, and Dankang Li

Supplementary Table S1. Comparison of Covariate Distributions Before and After Multiple Imputation

| Variable | Observed | Imputed | *P* |
| --- | --- | --- | --- |
| Age | 13.59 (1.64) | 13.68 (1.65) | <0.001 |
| Gender |  |  |  |
| Male | 444931 (48.4) | 442918 (48.2) | <0.001 |
| Female | 473633 (51.6) | 475646 (51.8) |  |
| School grade |  |  |  |
| Grade 5 | 247921 (32.9) | 281039 (30.6) | <0.001 |
| Grade 7 | 257256 (34.1) | 313678 (34.1) |  |
| Grade 9 | 248487 (33.0) | 323847 (35.3) |  |
| Physical activity |  |  |  |
| No Physical activity | 41189 (4.6) | 40978 ( 4.5) | <0.001 |
| 1 days | 67958 (7.6) | 69733 ( 7.6) |  |
| 2 days | 120389 (13.4) | 126794 (13.8) |  |
| 3 days | 149810 (16.7) | 156134 (17.0) |  |
| 4 days | 136790 (15.2) | 140868 (15.3) |  |
| 5 days | 129531 (14.4) | 131793 (14.3) |  |
| 6 days | 83612 (9.3) | 84266 ( 9.2) |  |
| 7 days | 168078 (18.7) | 167998 (18.3) |  |
| BMI | 19.49 (3.50) | 19.19 (3.30) | <0.001 |
| ﻿Family Affluence Scale |  |  |  |
| 0 | 8769 (1.0) | 9901 ( 1.1) | <0.001 |
| 1 | 22908 (2.6) | 26356 ( 2.9) |  |
| 2 | 45341 (5.1) | 51819 ( 5.6) |  |
| 3 | 77555 (8.8) | 86945 ( 9.5) |  |
| 4 | 115996 (13.1) | 127128 (13.8) |  |
| 5 | 147134 (16.7) | 156947 (17.1) |  |
| 6 | 163204 (18.5) | 168016 (18.3) |  |
| 7 | 140248 (15.9) | 140160 (15.3) |  |
| 8 | 97926 (11.1) | 93697 (10.2) |  |
| 9 | 64043 (7.3) | 57595 ( 6.3) |  |
| ﻿Academic pressure |  |  |  |
| Not at all | 185115 (20.4) | 179691 (19.6) | <0.001 |
| A little | 398606 (44.0) | 409820 (44.6) |  |
| Some | 217380 (24.0) | 228949 (24.9) |  |
| A lot | 104947 (11.6) | 100104 (10.9) |  |
| ﻿Experienced bullying |  |  |  |
| Never | 616492 (70.5) | 656591 (71.5) | <0.001 |
| Once or twice | 158947 (18.2) | 163214 (17.8) |  |
| 2-3 times per month | 39280 (4.5) | 38977 ( 4.2) |  |
| Once/week | 25472 (2.9) | 25465 ( 2.8) |  |
| Several times/week | 34534 (3.9) | 34316 ( 3.7) |  |
| Diet quality score |  |  | <0.001 |
| Poor | 496403 (54.4) | 501542 (54.8) |  |
| Intermediate | 269647 (29.5) | 272366 (29.8) |  |
| Ideal | 146871 (16.1) | 140761 (15.4) |  |

While P-values are <0.001 due to the extremely large sample size (N=918,564), the absolute differences in means and proportions are minimal, confirming the distributional stability after imputation.

Supplementary Table S2. Baseline Imbalance (Standardized Mean Differences, SMD) Between Included and Excluded Participants for Different Survey Year

| Variable | Overall | 2002 | 2006 | 2010 | 2014 | 2018 |
| --- | --- | --- | --- | --- | --- | --- |
| Age | 0.176 | 0.137 | 0.186 | 0.186 | 0.180 | 0.180 |
| Gender | 0.117 | 0.074 | 0.135 | 0.155 | 0.112 | 0.115 |
| School grade | 0.181 | 0.162 | 0.209 | 0.160 | 0.470 | 0.157 |
| Physical activity | 0.265 | 0.133 | 0.201 | 0.328 | 0.391 | 0.339 |
| BMI | 0.027 | 0.019 | 0.032 | 0.051 | 0.009 | 0.018 |
| Family Affluence Scale | 0.265 | 0.098 | 0.330 | 0.413 | 0.333 | 0.404 |
| Academic pressure | 0.387 | 0.114 | 0.312 | 0.370 | 0.653 | 0.336 |
| Experienced bullying | 0.322 | 0.546 | 0.240 | 0.346 | 0.361 | 0.220 |
| Diet quality score | 0.464 | 0.174 | 0.293 | 0.384 | 0.632 | 0.596 |
| Breakfast days | 0.056 | 0.006 | 0.013 | 0.060 | 0.118 | 0.129 |
| ﻿Psychosomatic complaints | 0.300 | 0.052 | 0.327 | 0.481 | 0.343 | 0.309 |

Abbreviations: BMI, body mass index; SMD, Standardized Mean Difference.

SMD thresholds: <0.10 negligible, 0.10-0.20 small, 0.20-0.50 moderate, >0.50 large.

Interpretation: Exposure (total breakfast days) shows negligible-small imbalance across all years (SMD≤0.129), suggesting limited selection bias on the exposure distribution. Larger SMDs appear mainly in lifestyle/psychosocial covariates; models adjust for these covariates and survey year.

Supplementary Table S3. Comparison of model fit for functional forms of breakfast frequency

| Model Specification | ΔAIC | ΔBIC |
| --- | --- | --- |
| Spline (GAM) | Ref. | Ref. |
| Quadratic | 1406.6 | 1350.7 |
| Linear | 1684.1 | 1617.0 |

All models were adjusted for the same set of covariates and used Restricted Maximum Likelihood (REML) for parameter estimation. ΔAIC and ΔBIC represent the difference between the candidate model and the best-fitting spline model.

Supplementary Table S4. Estimated degrees of freedom (EDF) for smooth terms in the final GAM

| **Smooth Term** | **Estimated Degrees of Freedom (EDF)** | **F statistic** | ***P*-value** |
| --- | --- | --- | --- |
| s(Breakfast days) | 6.87 | 1483.97 | < 0.001 |
| s(Age) | 3.73 | 40.09 | < 0.001 |
| s(BMI) | 4.65 | 111.84 | < 0.001 |
| s(Country ID) | 44.83 | 812.79 | < 0.001 |
| s(Survey Year) | 3.99 | 15163.26 | < 0.001 |

EDF > 1 indicates non-linearity. The EDF of 6.87 for breakfast frequency justifies using GAMs over simpler models to capture complex associations. Additionally, the high EDF for Country ID (~45) confirms that the model effectively accounts for cross-national heterogeneity within the HBSC multilevel structure.

Supplementary Table S5. Associations of Total Breakfast Days Categories with Psychosomatic ﻿Complaints Score Stratiﬁed by Gender

| Total Breakfast days | Boys | | Girls | | P for interaction (Breakfast × Gender) |
| --- | --- | --- | --- | --- | --- |
|  | β (95 CI) | *P* | β (95 CI) | *P* |  |
| 0 days | 2.02 (1.90-2.15) | <0.001 | 3.39 (3.26-3.52) | <0.001 | <0.001  (LRT χ^2^ = 191776.01, *P*<0.001) |
| 1 days | 1.91 (1.80-2.03) | <0.001 | 2.95 (2.85-3.06) | <0.001 |  |
| 2 days | 1.11 (1.04-1.18) | <0.001 | 1.74 (1.67-1.81) | <0.001 |  |
| 3 days | 1.31 (1.21-1.42) | <0.001 | 1.66 (1.55-1.76) | <0.001 |  |
| 4 days | 1.08 (0.98-1.17) | <0.001 | 1.64 (1.54-1.74) | <0.001 |  |
| 5 days | 0.96 (0.88-1.04) | <0.001 | 1.34 (1.25-1.42) | <0.001 |  |
| 6 days | 0.78 (0.71-0.85) | <0.001 | 0.98 (0.90-1.06) | <0.001 |  |
| 7 days | Ref. |  | Ref. |  |  |
| *P*-trend | <0.001 |  | <0.001 |  |  |

Abbreviations: CI, confidence interval; Ref, reference.

The βs and 95 CIs were extracted from multilevel generalized additive model (GAM), with psychosomatic complaints score as the outcome and variable of total breakfast days categories as the exposure.

The 7 days category (daily breakfast) was used as the reference.

*P* value for trend calculated treating the total breakfast days as a continuous variable.

*P* for interaction (Breakfast × Gender) was obtained from the fully adjusted model including the interaction term between total breakfast days categories and gender, evaluated using a Likelihood Ratio Test.

Models were adjusted for ﻿survey year (only for overall participants), school grade, physical activity, Family Affluence Scale, academic pressure, having experienced bullying, diet quality score, and smooth term of age and BMI.

Supplementary Table S6. Associations of Total Breakfast Days Categories with Psychosomatic ﻿Complaints Score Stratiﬁed by School Grade

| Total Breakfast days | School Grade 5 | | School Grade 7 | | School Grade 9 | | P for interaction  (Breakfast × School Grade) |
| --- | --- | --- | --- | --- | --- | --- | --- |
|  | β (95 CI) | *P* | β (95 CI) | *P* | β (95 CI) | *P* |  |
| 0 days | 2.07 (1.86-2.28) | <0.001 | 2.88 (2.75-3.01) | <0.001 | 2.98 (2.82-3.14) | <0.001 | <0.001  (LRT χ^2^ = 6556.98, *P*<0.001) |
| 1 days | 2.17 (2.00-2.35) | <0.001 | 2.59 (2.47-2.70) | <0.001 | 2.68 (2.55-2.81) | <0.001 |  |
| 2 days | 1.14 (1.04-1.24) | <0.001 | 1.55 (1.47-1.63) | <0.001 | 1.60 (1.51-1.68) | <0.001 |  |
| 3 days | 1.16 (1.02-1.30) | <0.001 | 1.68 (1.56-1.81) | <0.001 | 1.57 (1.44-1.70) | <0.001 |  |
| 4 days | 1.11 (0.97-1.24) | <0.001 | 1.58 (1.47-1.70) | <0.001 | 1.39 (1.27-1.51) | <0.001 |  |
| 5 days | 0.91 (0.80-1.02) | <0.001 | 1.24 (1.15-1.34) | <0.001 | 1.27 (1.17-1.37) | <0.001 |  |
| 6 days | 0.83 (0.73-0.93) | <0.001 | 0.88 (0.78-0.97) | <0.001 | 0.96 (0.86-1.05) | <0.001 |  |
| 7 days | Ref. |  | Ref. |  | Ref. |  |  |
| *P*-trend | <0.001 |  | <0.001 |  | <0.001 |  |  |

Abbreviations: CI, confidence interval; Ref, reference.

The βs and 95 CIs were extracted from multilevel generalized additive model (GAM), with psychosomatic complaints score as the outcome and variable of total breakfast days categories as the exposure.

The 7 days category (daily breakfast) was used as the reference.

*P* value for trend calculated treating the total breakfast days as a continuous variable.

*P* for interaction (Breakfast × School Grade) was obtained from the fully adjusted model including the interaction term between total breakfast days categories and school grade, evaluated using a Likelihood Ratio Test.

Models were adjusted for ﻿survey year (only for overall participants), gender, physical activity, Family Affluence Scale, academic pressure, having experienced bullying, diet quality score, and smooth term of age and BMI.

Supplementary Table S7. Associations of Total Breakfast Days Categories with Psychosomatic ﻿Complaints Score After Restricting to Participants Who with Complete Covariate Information to Minimize Potential Bias from Missing Data

| Total Breakfast days | Overall | | Survey 2002 | | Survey 2006 | | Survey 2010 | | Survey 2014 | | Survey 2018 | |
| --- | --- | --- | --- | --- | --- | --- | --- | --- | --- | --- | --- | --- |
|  | β（95 CI） | *P* | β（95 CI） | *P* | β（95 CI） | *P* | β（95 CI） | *P* | β（95 CI） | *P* | β（95 CI） | *P* |
| 0 days | 2.86 (2.76-2.95) | <0.001 | 2.59 (2.37-2.81) | <0.001 | 2.76 (2.56-2.95) | <0.001 | 2.57 (2.36-2.78) | <0.001 | 2.93 (2.70-3.16) | <0.001 | 2.99 (2.80-3.18) | <0.001 |
| 1 days | 2.63 (2.55-2.71) | <0.001 | 2.26 (2.08-2.44) | <0.001 | 2.40 (2.24-2.57) | <0.001 | 2.53 (2.35-2.71) | <0.001 | 2.63 (2.43-2.84) | <0.001 | 2.96 (2.79-3.12) | <0.001 |
| 2 days | 1.51 (1.46-1.56) | <0.001 | 1.36 (1.25-1.47) | <0.001 | 1.48 (1.38-1.58) | <0.001 | 1.42 (1.31-1.54) | <0.001 | 1.51 (1.38-1.63) | <0.001 | 1.61 (1.50-1.72) | <0.001 |
| 3 days | 1.58 (1.50-1.66) | <0.001 | 1.35 (1.17-1.53) | <0.001 | 1.63 (1.47-1.79) | <0.001 | 1.69 (1.52-1.87) | <0.001 | 1.63 (1.43-1.83) | <0.001 | 1.39 (1.23-1.55) | <0.001 |
| 4 days | 1.44 (1.37-1.51) | <0.001 | 1.16 (1.00-1.33) | <0.001 | 1.46 (1.31-1.60) | <0.001 | 1.37 (1.20-1.53) | <0.001 | 1.64 (1.46-1.82) | <0.001 | 1.41 (1.26-1.56) | <0.001 |
| 5 days | 1.20 (1.14-1.26) | <0.001 | 1.06 (0.92-1.19) | <0.001 | 1.32 (1.20-1.43) | <0.001 | 1.09 (0.96-1.22) | <0.001 | 1.24 (1.09-1.38) | <0.001 | 1.14 (1.02-1.27) | <0.001 |
| 6 days | 0.92 (0.86-0.97) | <0.001 | 0.77 (0.65-0.89) | <0.001 | 0.92 (0.81-1.03) | <0.001 | 1.05 (0.92-1.17) | <0.001 | 0.93 (0.79-1.07) | <0.001 | 0.83 (0.71-0.95) | <0.001 |
| 7 days | Ref. |  | Ref. |  | Ref. |  | Ref. |  | Ref. |  | Ref. |  |
| *P*-trend | <0.001 |  | <0.001 |  | <0.001 |  | <0.001 |  | <0.001 |  | <0.001 |  |

﻿Abbreviations: CI, confidence interval; Ref, reference.

The βs and 95 CIs were extracted from multilevel generalized additive model (GAM), with psychosomatic complaints as the outcome and variable of total breakfast days categories as the exposure.

The 7 days category (daily breakfast) was used as the reference.

*P* value for trend calculated treating the total breakfast days as a continuous variable.

Models were adjusted for survey year (only for overall participants), gender, school grade, physical activity, Family Affluence Scale, academic pressure, having experienced bullying, diet quality score, easy to talk with father, easy to talk with mother, and smooth term of age and BMI.

Supplementary Table S8. Associations of Total Breakfast Days Categories with Psychosomatic ﻿Complaints Score after Further Adjusting Easy to Talk with Father and Easy to Talk with Mother

| Total Breakfast days | Overall | | Survey 2002 | | Survey 2006 | | Survey 2010 | | Survey 2014 | | Survey 2018 | |
| --- | --- | --- | --- | --- | --- | --- | --- | --- | --- | --- | --- | --- |
|  | β（95 CI） | *P* | β（95 CI） | *P* | β（95 CI） | *P* | β（95 CI） | *P* | β（95 CI） | *P* | β（95 CI） | *P* |
| 0 days | 2.51 (2.42-2.60) | <0.001 | 2.19 (1.98-2.40) | <0.001 | 2.39 (2.20-2.58) | <0.001 | 2.33 (2.11-2.55) | <0.001 | 2.62 (2.39-2.85) | <0.001 | 2.55 (2.36-2.74) | <0.001 |
| 1 days | 2.33 (2.25-2.41) | <0.001 | 1.95 (1.78-2.12) | <0.001 | 2.04 (1.88-2.20) | <0.001 | 2.37 (2.18-2.55) | <0.001 | 2.33 (2.13-2.54) | <0.001 | 2.60 (2.43-2.76) | <0.001 |
| 2 days | 1.38 (1.33-1.43) | <0.001 | 1.20 (1.09-1.31) | <0.001 | 1.30 (1.20-1.40) | <0.001 | 1.53 (1.41-1.65) | <0.001 | 1.31 (1.18-1.44) | <0.001 | 1.37 (1.26-1.48) | <0.001 |
| 3 days | 1.44 (1.36-1.52) | <0.001 | 1.20 (1.02-1.37) | <0.001 | 1.45 (1.29-1.60) | <0.001 | 1.64 (1.46-1.82) | <0.001 | 1.46 (1.27-1.66) | <0.001 | 1.20 (1.04-1.36) | <0.001 |
| 4 days | 1.33 (1.26-1.40) | <0.001 | 1.07 (0.91-1.23) | <0.001 | 1.32 (1.18-1.47) | <0.001 | 1.30 (1.13-1.46) | <0.001 | 1.51 (1.33-1.69) | <0.001 | 1.24 (1.09-1.39) | <0.001 |
| 5 days | 1.10 (1.04-1.15) | <0.001 | 0.92 (0.79-1.05) | <0.001 | 1.13 (1.02-1.25) | <0.001 | 1.07 (0.94-1.21) | <0.001 | 1.12 (0.98-1.27) | <0.001 | 1.04 (0.91-1.17) | <0.001 |
| 6 days | 0.83 (0.77-0.88) | <0.001 | 0.64 (0.52-0.76) | <0.001 | 0.79 (0.68-0.89) | <0.001 | 1.02 (0.90-1.15) | <0.001 | 0.86 (0.72-1.00) | <0.001 | 0.71 (0.59-0.84) | <0.001 |
| 7 days | Ref. |  | Ref. |  | Ref. |  | Ref. |  | Ref. |  | Ref. |  |
| *P*-trend | <0.001 |  | <0.001 |  | <0.001 |  | <0.001 |  | <0.001 |  | <0.001 |  |

﻿Abbreviations: CI, confidence interval; Ref, reference.

The βs and 95 CIs were extracted from multilevel generalized additive model (GAM), with psychosomatic complaints as the outcome and variable of total breakfast days categories as the exposure.

The 7 days category (daily breakfast) was used as the reference.

*P* value for trend calculated treating the total breakfast days as a continuous variable.

Models were adjusted for ﻿survey year (only for overall participants), gender, school grade, physical activity, Family Affluence Scale, academic pressure, having experienced bullying, diet quality score, easy to talk with father, easy to talk with mother, and smooth term of age and BMI.

Supplementary Table S9. Associations of Total Breakfast Days Categories with Psychosomatic ﻿Complaints Score for Survey 2014 and Survey 2018

| Total Breakfast days | Overall (2014 and 2018) | | Survey 2014 | | Survey 2018 | |
| --- | --- | --- | --- | --- | --- | --- |
|  | β（95 CI） | *P* | β（95 CI） | *P* | β（95 CI） | *P* |
| 0 days | 2.40 (2.26-2.55) | <0.001 | 2.44 (2.21-2.67) | <0.001 | 2.38 (2.19-2.57) | <0.001 |
| 1 days | 2.33 (2.20-2.46) | <0.001 | 2.19 (1.98-2.40) | <0.001 | 2.40 (2.23-2.57) | <0.001 |
| 2 days | 1.25 (1.17-1.34) | <0.001 | 1.25 (1.12-1.38) | <0.001 | 1.25 (1.14-1.37) | <0.001 |
| 3 days | 1.19 (1.07-1.32) | <0.001 | 1.31 (1.11-1.51) | <0.001 | 1.11 (0.95-1.27) | <0.001 |
| 4 days | 1.22 (1.10-1.33) | <0.001 | 1.36 (1.18-1.54) | <0.001 | 1.11 (0.96-1.26) | <0.001 |
| 5 days | 0.94 (0.85-1.04) | <0.001 | 1.01 (0.86-1.15) | <0.001 | 0.90 (0.77-1.03) | <0.001 |
| 6 days | 0.66 (0.56-0.75) | <0.001 | 0.73 (0.59-0.87) | <0.001 | 0.59 (0.47-0.72) | <0.001 |
| 7 days | Ref. |  | Ref. |  | Ref. |  |
| *P*-trend | <0.001 |  | <0.001 |  | <0.001 |  |

﻿Abbreviations: CI, confidence interval; Ref, reference.

The βs and 95 CIs were extracted from multilevel generalized additive model (GAM), with psychosomatic complaints score as the outcome and variable of total breakfast days categories as the exposure.

The 7 days category (daily breakfast) was used as the reference.

*P* value for trend calculated treating the total breakfast days as a continuous variable.

Models were adjusted for ﻿survey year (only for overall participants), gender, school grade, physical activity, Family Affluence Scale, academic pressure, having experienced bullying, diet quality score, easy to talk with father, easy to talk with mother, family support, and smooth term of age and BMI.

Supplementary Table S10. Associations of Total Breakfast Days Categories with Psychosomatic ﻿Complaints Score after using multiple imputation with fully conditional specification

| Total Breakfast days | Overall | | Survey 2002 | | Survey 2006 | | Survey 2010 | | Survey 2014 | | Survey 2018 | |
| --- | --- | --- | --- | --- | --- | --- | --- | --- | --- | --- | --- | --- |
|  | β（95 CI） | *P* | β（95 CI） | *P* | β（95 CI） | *P* | β（95 CI） | *P* | β（95 CI） | *P* | β（95 CI） | *P* |
| 0 days | 3.07 (3.00-3.14) | <0.001 | 2.62 (2.43-2.80) | <0.001 | 2.77 (2.61-2.93) | <0.001 | 2.92 (2.76-3.08) | <0.001 | 3.12 (2.95-3.28) | <0.001 | 3.24 (3.11-3.38) | <0.001 |
| 1 days | 2.73 (2.67-2.80) | <0.001 | 2.28 (2.12-2.43) | <0.001 | 2.45 (2.32-2.59) | <0.001 | 2.66 (2.52-2.80) | <0.001 | 2.81 (2.66-2.95) | <0.001 | 2.99 (2.87-3.11) | <0.001 |
| 2 days | 1.62 (1.58-1.66) | <0.001 | 1.39 (1.29-1.49) | <0.001 | 1.50 (1.41-1.59) | <0.001 | 1.62 (1.53-1.71) | <0.001 | 1.70 (1.61-1.80) | <0.001 | 1.67 (1.58-1.76) | <0.001 |
| 3 days | 1.63 (1.57-1.69) | <0.001 | 1.34 (1.18-1.49) | <0.001 | 1.66 (1.52-1.80) | <0.001 | 1.73 (1.60-1.86) | <0.001 | 1.66 (1.52-1.80) | <0.001 | 1.50 (1.37-1.62) | <0.001 |
| 4 days | 1.49 (1.43-1.55) | <0.001 | 1.22 (1.08-1.37) | <0.001 | 1.44 (1.32-1.57) | <0.001 | 1.46 (1.34-1.59) | <0.001 | 1.62 (1.49-1.75) | <0.001 | 1.43 (1.31-1.54) | <0.001 |
| 5 days | 1.29 (1.24-1.34) | <0.001 | 1.14 (1.02-1.25) | <0.001 | 1.33 (1.23-1.43) | <0.001 | 1.23 (1.13-1.33) | <0.001 | 1.37 (1.26-1.48) | <0.001 | 1.20 (1.10-1.30) | <0.001 |
| 6 days | 0.95 (0.90-0.99) | <0.001 | 0.78 (0.67-0.89) | <0.001 | 0.89 (0.79-0.98) | <0.001 | 1.03 (0.93-1.12) | <0.001 | 0.99 (0.89-1.09) | <0.001 | 0.90 (0.80-0.99) | <0.001 |
| 7 days | Ref. |  | Ref. |  | Ref. |  | Ref. |  | Ref. |  | Ref. |  |
| *P*-trend | <0.001 |  | <0.001 |  | <0.001 |  | <0.001 |  | <0.001 |  | <0.001 |  |

﻿Abbreviations: CI, confidence interval; Ref, reference.

The βs and 95 CIs were extracted from multilevel generalized additive model (GAM), with psychosomatic complaints as the outcome and variable of total breakfast days categories as the exposure.

The 7 days category (daily breakfast) was used as the reference.

*P* value for trend calculated treating the total breakfast days as a continuous variable.

Models were adjusted for ﻿survey year (only for overall participants), gender, school grade, physical activity, Family Affluence Scale, academic pressure, having experienced bullying, diet quality score, easy to talk with father, easy to talk with mother, and smooth term of age and BMI.

Supplementary Table S11. Associations of Total Breakfast Days Categories with Psychosomatic ﻿Complaints Score adopted a cross-validation approach by dividing the dataset into 5 folds

| Total Breakfast days | Sub dataset 1 | | Sub dataset 2 | | Sub dataset 3 | | Sub dataset 4 | | Sub dataset 5 | |
| --- | --- | --- | --- | --- | --- | --- | --- | --- | --- | --- |
|  | β (95 CI) | *P* | β (95 CI) | *P* | β (95 CI) | *P* | β (95 CI) | *P* | β (95 CI) | *P* |
| 0 days | 2.80 (2.70-2.90) | <0.001 | 2.78 (2.68-2.88) | <0.001 | 2.76 (2.65-2.86) | <0.001 | 2.78 (2.67-2.88) | <0.001 | 2.79 (2.69-2.89) | <0.001 |
| 1 days | 2.59 (2.50-2.68) | <0.001 | 2.52 (2.43-2.61) | <0.001 | 2.55 (2.47-2.64) | <0.001 | 2.58 (2.49-2.66) | <0.001 | 2.59 (2.50-2.67) | <0.001 |
| 2 days | 1.48 (1.42-1.53) | <0.001 | 1.49 (1.43-1.54) | <0.001 | 1.46 (1.41-1.52) | <0.001 | 1.48 (1.43-1.54) | <0.001 | 1.48 (1.42-1.53) | <0.001 |
| 3 days | 1.54 (1.45-1.62) | <0.001 | 1.54 (1.46-1.63) | <0.001 | 1.55 (1.46-1.63) | <0.001 | 1.46 (1.38-1.55) | <0.001 | 1.51 (1.43-1.60) | <0.001 |
| 4 days | 1.41 (1.33-1.49) | <0.001 | 1.39 (1.31-1.47) | <0.001 | 1.42 (1.34-1.50) | <0.001 | 1.42 (1.34-1.50) | <0.001 | 1.38 (1.30-1.46) | <0.001 |
| 5 days | 1.19 (1.13-1.26) | <0.001 | 1.16 (1.10-1.23) | <0.001 | 1.16 (1.10-1.23) | <0.001 | 1.16 (1.09-1.22) | <0.001 | 1.17 (1.11-1.24) | <0.001 |
| 6 days | 0.90 (0.84-0.96) | <0.001 | 0.90 (0.84-0.96) | <0.001 | 0.90 (0.84-0.96) | <0.001 | 0.90 (0.84-0.97) | <0.001 | 0.88 (0.82-0.94) | <0.001 |
| 7 days | Ref. |  | Ref. |  | Ref. |  | Ref. |  | Ref. |  |
| *P*-trend | <0.001 |  | <0.001 |  | <0.001 |  | <0.001 |  | <0.001 |  |

Abbreviations: CI, confidence interval; Ref, reference; Sub, subsets.

Five-fold cross-validation by randomly partitioning the dataset into five subsets.

The βs and 95 CIs were extracted from multilevel generalized additive model (GAM), with psychosomatic complaints score as the outcome and variable of total breakfast days categories as the exposure.

The 7 days category (daily breakfast) was used as the reference.

*P* value for trend calculated treating the total breakfast days as a continuous variable.

Models were adjusted for ﻿survey year (only for overall participants), gender, school grade, physical activity, Family Affluence Scale, academic pressure, having experienced bullying, diet quality score, and smooth term of age and BMI.


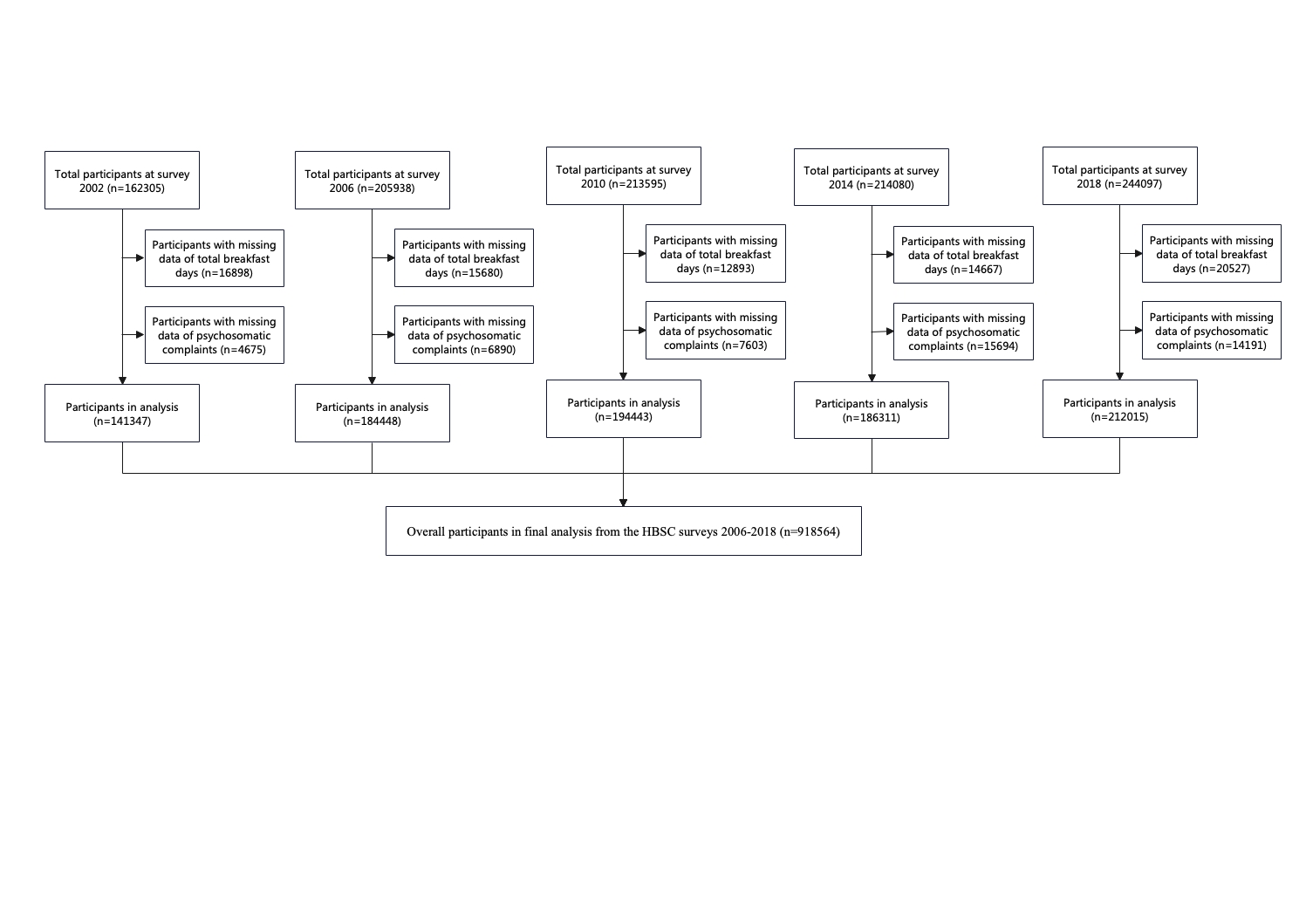


Supplementary Figure S1. Flowchart of the Participants in the Present Study.


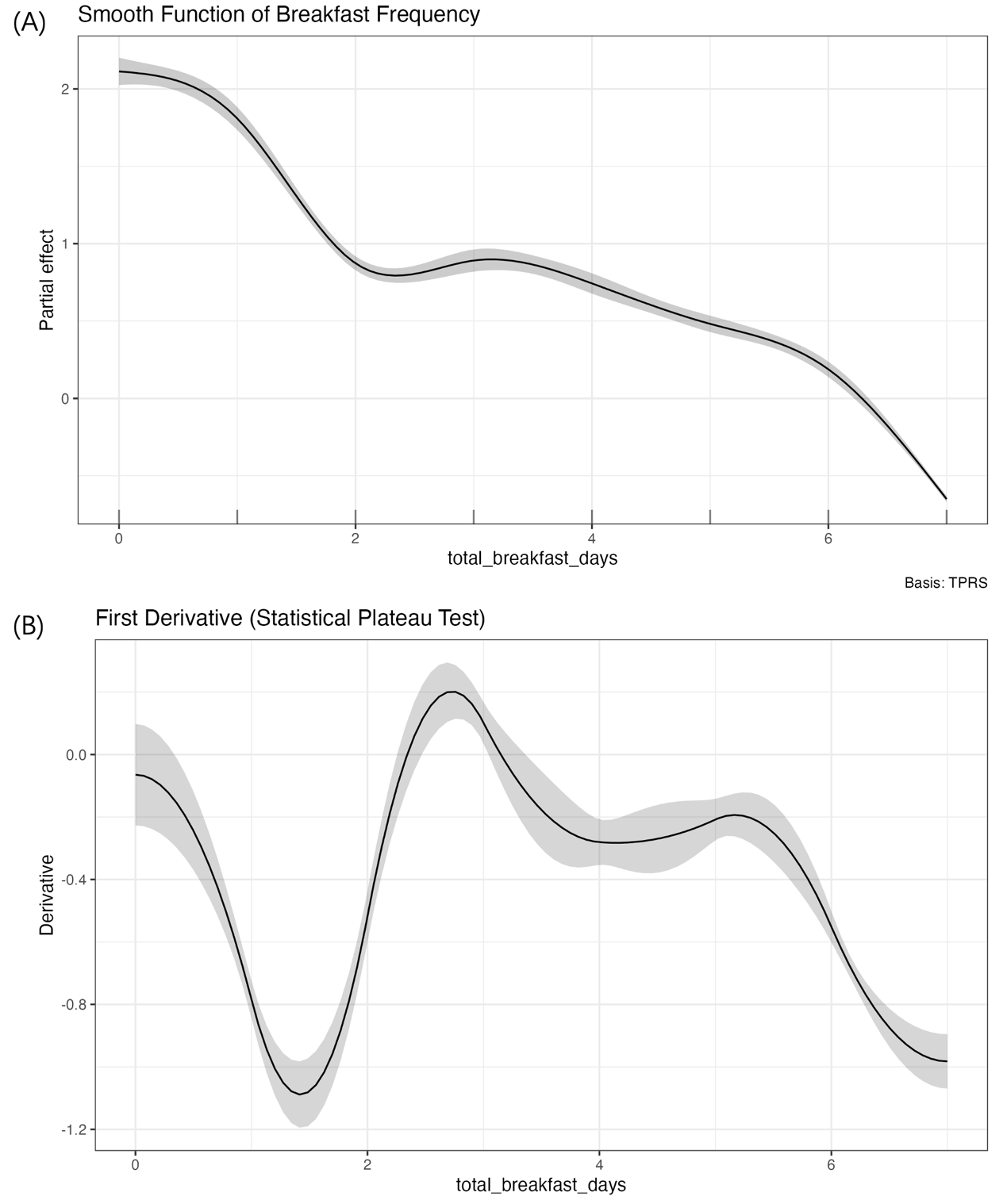


Supplementary Figure S2. Smooth Function and First-Derivative Analysis. (A) Smooth regression spline of breakfast frequency on psychosomatic complaints score, with the shaded area indicating the 95% Bayesian credible interval. (B) First derivative of the smooth function. A statistical plateau is identified where the 95% confidence interval of the derivative includes zero, indicating no significant marginal change in the outcome.


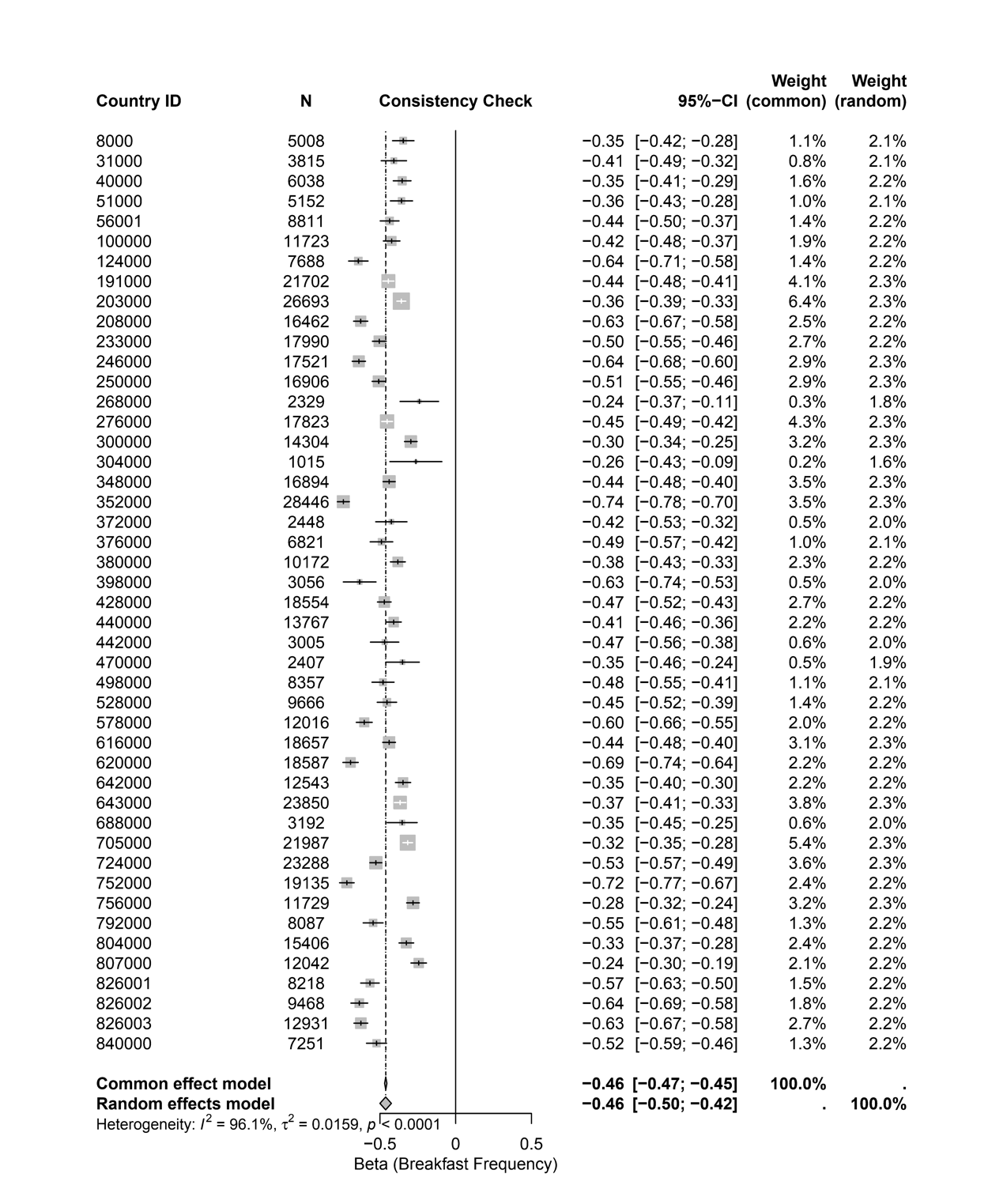


Supplementary Figure S3. Forest plot of the association between breakfast frequency and psychosomatic complaints across different countries/regions.

Each point represents the adjusted beta coefficient from country-specific weighted linear regression, and the horizontal lines represent the 95% confidence intervals. The overall pooled direction demonstrates a consistent inverse association across all included settings, supporting the robustness of the global trend.


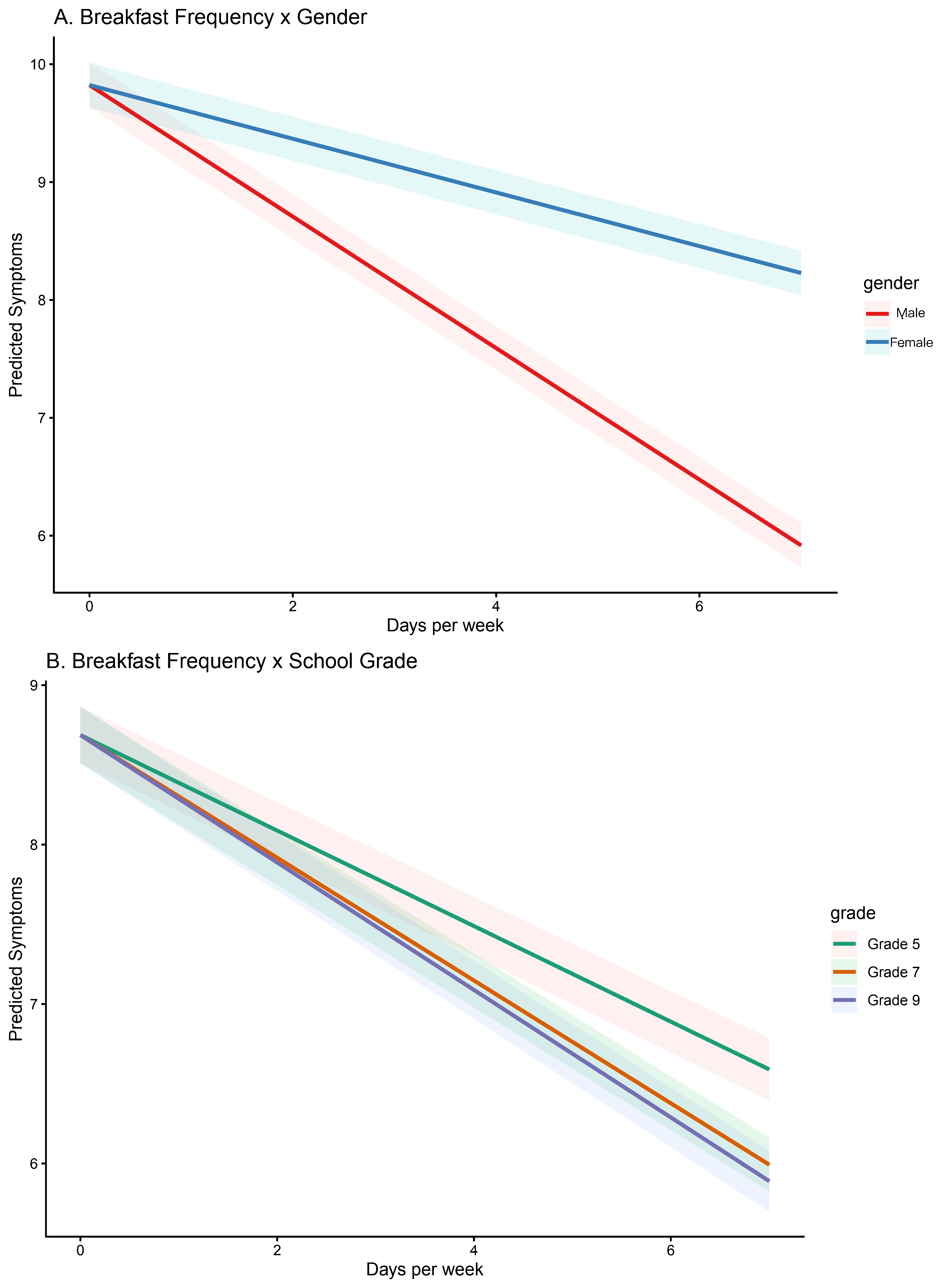


Supplementary Figure S4. Interaction plots of breakfast frequency with gender and school grade on psychosomatic complaints scores. (A) Interaction between breakfast frequency and gender. (B) Interaction between breakfast frequency and school grade. Separate lines are provided for Grade 5, Grade 7, and Grade 9. Adjusted predicted scores were estimated using multilevel generalized additive models (GAMs). Solid lines indicate the predicted mean psychosomatic complaints scores, and shaded areas represent the 95% Bayesian credible intervals. For both gender and school grade, the interaction terms were statistically significant (both P-interaction​<0.001), evaluated using the Likelihood Ratio Test (LRT).

Supplementary Method

# Technical Appendix A: Reproducible Statistical Framework

# 1. Weight Normalization

data_analytic <- data_all %>%

group_by(Country.number, Survey.year) %>%

mutate(weight_norm = HBSC.weights / mean(HBSC.weights, na.rm = TRUE)) %>%

ungroup()

# 2. Multilevel GAM Specification

m_final <- gam(

Mental_Health_Score ~

s(total_breakfast_days, k=8, bs="tp") +

s(age, k=6, bs="tp") +

s(BMI, k=6, bs="tp") +

gender + Physical.activity + School.grade +

Family.Affluence.Scale + Academic.pressure +

Experienced.bullying + Diet.score.cater +

s(Country.number, bs="re") +

s(Survey.year, bs="re"),

data = data_analytic,

weights = weight_norm,

method = "REML"

)

# 3. Variance Components and ICC Calculation

v_comp <- gam.vcomp(m_final)

std_country <- as.numeric(v_comp[grep("Country.number", names(v_comp))])

std_year <- as.numeric(v_comp[grep("Survey.year", names(v_comp))])

var_residual <- as.numeric(m_final$sig2) # Residual Variance = 33.7187

icc_country <- (std_country^2) / (std_country^2 + std_year^2 + var_residual)

print(paste("Country-level ICC:", round(icc_country, 6)))
